# Systemic levels of iron, phosphorus, and total protein in normocyclic versus repeat breeder Holstein Friesian crossbred cows of Kesharbag, Chitwan, Nepal

**DOI:** 10.14202/vetworld.2020.2353-2357

**Published:** 2020-11-06

**Authors:** Girija Regmi, Ishwari Prasad Dhakal

**Affiliations:** 1 Oklahoma Medical Research Foundation, Oklahoma City, Oklahoma, USA; 2 Institute of Agriculture and Animal Sciences, Agriculture and Forestry University, Rampur, Chitwan, Nepal

**Keywords:** crossbred cows, estrous cyclicity, normocyclic, repeat breeder/breeding

## Abstract

**Background and Aim:**

In repeat breeding, a sexually mature cow fails to conceive even after three or more consecutive inseminations despite being without any clinically detectable reproductive anomalies. This is a major cause of economic loss in livestock farms, particularly in developing countries, where humans and livestock directly compete for food, and the mineral content of animal feed is rarely checked. This study investigated the association between systemic iron, phosphorus, and total protein and estrous cyclicity in crossbred Holstein Friesian cows.

**Materials and Methods:**

Blood samples were collected from 10 normal cyclic and 10 repeat breeder cows 12 h after the onset of estrus. Serum was separated, and iron, phosphorus, and total protein were quantified with spectrophotometry, using standard controls for all three measurement parameters (iron, phosphorus, and total protein).

**Results:**

Iron and phosphorus levels were significantly (p<0.05) lower in the repeat breeders group than in the normocyclic group, but no significant differences were found in total protein levels.

**Conclusion:**

Repeat breeding is associated with systemic iron and phosphorus levels but is independent of total protein level.

## Introduction

A repeat breeder cow fails to conceive even after three or more successive inseminations in the absence of clinically detectable reproductive anomalies [1]. Repeat breeding is a common problem in dairy animals and results in significant economic loss [2]. It is the second most common cause of economic loss in farms where livestock are raised for milk [3]. Any change in the rhythm of regular breeding causes significant economic loss in multiple ways, including decreased lactation periods, reduced calving, long dry periods, increased in herd maintenance costs due to multiple replacement costs, and the cost of semen and of insemination [4]. Understanding the underlying causes of repeat breeding can help minimize its incidence in dairy farms and subsequently reduce the economic loss associated with it. A multitude of factors can lead to repeat breeding, such as failure of fertilization, early embryonic death, congenital or genetic defects of the genital tract, congenital or acquired defects of the ova, spermatozoa, or early zygote, infectious or traumatic inflammation, endocrine/hormonal dysfunction, and managerial deficiencies (including failure to identify estrus) [5].

Because trace mineral ions such as Fe, Ca, and Zn act as cofactors for different enzymes responsible for nucleic acid, proteins, and carbohydrate metabolism, change in the level of these minerals may affect hormone production, hormone activity, ovarian function, and the constituents of reproduction tract secretion, consequently impacting reproductive health [5]. Nutritional deficiencies or imbalances may be among the major causes of repeat breeding in developing countries such as Nepal, where there is direct competition for food between humans and livestock and the status of soil minerals and minerals in diet is rarely checked [6]. Among these minerals, selenium, calcium, zinc, copper, and manganese have been extensively studied, but few studies have investigated the role of total serum proteins (albumin and globulins), iron, and phosphorus in Southeast Asia, particularly in Nepal.

This study investigated the association between repeat breeding and levels of iron, phosphorus, and total protein in the serum of Holstein Friesian crossbred cows in this region. We hypothesize that variation in the systemic level of iron, phosphorus, and total protein may contribute to repeat breeding in these cows.

## Materials and Methods

### Ethical approval

This study was conducted with the ethical approval from Chitwan District Livestock Service Office and University Ethics and Authority board of the Institute of Agriculture and Animal Sciences (IAAS), Rampur.

### Animals

A control group of 10 normal crossbred Holstein Friesian cows and a study group of 10 repeat breeder Holstein cows with an age range of 3-8 years and a parity range of 0-4, at a point in the lactation period from late to terminal lactation, close to the dry period, and with a body condition score range of 2-5 were selected from private farms of Kesharbag, Chitwan, Nepal. All animals were raised under stall-fed (sharing a diet from the local community dairy center) conditions with water *ad libitum*. Both groups were chosen based on breeding history and clinical signs observed by experienced veterinarians. The absence of uterine, cervical, and vaginal inflammation/infection was confirmed, as well as ovarian pathologies such as ovarian cysts, for all of the animals under study.

The control group cows were apparently healthy and had normal estrous cyclicity, but the repeat breeder group failed to conceive during at least three successive attempts of proper artificial insemination or mating with a fertile bull during estrus between 4 and 12 h after the onset of standing heat. Other apparent diseases or abnormalities and all apparent causes of conception failure were ruled by an experienced veterinarian who performed per rectal examination of uteri and ovaries. The detection of estrus in both groups was performed twice a day (morning and evening) through visual observation. Once the animals were confirmed in standing heat, they were served with a fertile bull or were artificially inseminated 12 h after the onset of estrus. Pregnancy diagnoses were confirmed by rectal palpation and checking for fetal membrane slip and/or fremitus in all animals.

### Study site and season

This study was conducted in the tropical Chitwan Valley from the beginning of winter (December) to the end of spring (March), when there was very low heat stress to the cows.. The study period was mainly cool, dry, and less humid, with some scattered rainfall. The study area is located at a latitude of 27° 40’ 59.99” N, a longitude of 84° 25’ 59.99” E, and an altitude of 682 m above sea level and receives an annual rainfall of 1993 mm.

### Sample collection

Blood samples were drawn from the jugular vein in yellow BD Vacutainer glass tubes after proper restraint. The serum was separated by allowing the full tubes to stand at 22°C for 30 min and then centrifuged for 10 min at 1000 g. Upper straw-colored serum was collected in a sterile vial and kept at −20°C until the analyses.

### Measurement of iron, phosphorus, and total protein

The levels of iron, phosphorus, and total protein were measured spectrophotometrically, following the manufacturer’s instructions for the kits used (Direct Total Iron colorimetric assay for Serum/Plasma Cat. # 1230 [96 tests], Life Technologies [India] Pvt. Ltd., Phosphate Assay Kit [Colorimetric] Cat. # ab65622, Abcam [India]).

### Statistical analyses

The means (±standard error) for different components (total iron, phosphorus, and total protein) were computed. The data were analyzed using Student’s t-test to determine significant differences from the controls at p<0.05.

## Results and Discussion

Age, parity, lactation period, and body condition scores for the respective normocyclic and repeat breeder cows in this study are given in Tables-1-4, respectively.

**Table-1:** Age of normocyclic and repeat breeder cows.

Age (years)	Normocyclic	Repeat breeder
3	2	3
4	4	1
5	1	3
6	2	0
7	1	2
8	0	1

**Table-2:** Parity of normocyclic and repeat breeder cows.

Parity	Normocyclic	Repeat breeder
Heifers (0)	2	3
1-2	5	4
3-4	3	3

**Table-3:** Lactation stage of normocyclic and repeat breeder cows.

Months after calving	Normocyclic	Repeat breeder
6-8	2	1
8-10	2	3
10-12	4	3

**Table-4:** BCS of normocyclic and repeat breeder cows.

BCS	Normocyclic	Repeat breeder
2-2.5	1	2
2.5-3.0	2	4
3.0-3.5	2	3
3.5-4.0	3	1
4.0-4.5	2	0

BCS=Body condition score

Levels of iron and phosphorus in serum were significantly lower (p<0.05) in the repeat breeder group than in the control group, and the levels of total protein were comparable between the two groups, with a non-significant (p<0.05) difference (Table-5). Modi *et al*. [7] had similar findings to those of our study, namely, of significantly lower levels of iron and estral discharge fluid. Rupde *et al*. [8] also reported lower levels of iron in repeat breeder animals than in normocyclic cows, but the difference in iron content from that of the control cows was non-significant. This difference in the results may be due to the effect of variation in breed, diet, and/or iron absorption efficiency, as iron is an oxygen carrier. Rupde *et al*. [8] and Jain [9] showed that low blood levels of iron result in iron deficiency anemia, which may impair the implantation and survival of embryos, as low iron contents decrease the osmolality of the oviductal fluid, causing embryonic death. Tiwari *et al*. [10] also reported lower systemic iron levels in repeat breeder buffaloes than in normocyclic ones.

**Table-5:** Systemic iron, phosphorus, and total protein level in normocyclic and repeat breeder cows.

Blood serum constituents	Repeat breeder (Mean±SE) n=10	Normocyclic (Mean±SE) n=10
Phosphorus (mg/100 mL)	5.91±0.51* (2.17-10.83)	6.24±0.28 (4.84-7.45)
Total protein (g/100 mL)	6.95 ±0.18 (5.1-8.92)	6.38±0.15 (6.12-7.53)
Iron (µmol/L)	18.33±1.01* (7.58-26.23)	22.74±0.82 (17.73-26.15)

* Significant, p<0.05; NS=Non-significant (p<0.05). Values in parentheses indicate ranges. SE=Standard error

Burle *et al*. [11] found significantly lower levels of inorganic phosphorus in repeat breeder sera compared to normocyclic animal sera, which is consistent with our current finding. Likewise, other studies [10,12,13] have reported lower serum levels of inorganic phosphorous in repeat breeder buffaloes than in normocyclic ones. Guzel *et al*. [14] observed no significant difference in total protein level in the sera of repeat breeder cows compared to normocyclic cows. Contrary to these findings and in contrast with our results, Burle *et al*. [11] reported a significant increase in total serum protein in cyclic cows relative to non-cyclic cows. In addition, Savalia *et al*. [15] observed lower levels of total protein in normal cyclic buffaloes than in a repeat breeder group, and Barui *et al*. [16] reported no significant difference in the total levels of inorganic phosphorous in repeat breeders relative to normocyclic buffaloes. This variation in results demands focused studies of the calcium/phosphorus ratio, the albumin/globulin ratio, and their turnover, not just static systemic levels that can better depict the health status and thereby the reproductive efficiency of the animal. Although there is a difference in the circulating levels of certain biochemicals between normocyclic and repeat breeder cows, there are a multitude of other factors as well, such as genetic factors, genital heath, and breeding management, which may contribute to repeat breeding, should also be considered to reduce the incidence of repeat breeding [17].

Our results suggest that the total dietary protein content was comparable between the repeat breeder and normocyclic cows. This result rules out the possibility that protein deficiency alone could be directly correlated with the problem of repeat breeding. At the same time, significantly lower levels of iron and phosphorus in serum may be linked to the problem of repeat breeding. It may indicate either that the diet is deficient in iron and phosphorus or that there is a problem with diminished absorption or increased clearance. Although we cannot assert that iron and phosphorus deficiency are the main cause of repeat breeding in cows, it may be associated with it.

Levels of iron and phosphorus were significantly lower in crossbred repeat breeder Holstein Friesian cows from Chitwan Valley, and differences in levels of total protein were non-significant. This finding may help clarify the relationship of repeat breeding to mineral levels in blood, particularly levels of iron and phosphorus in serum. Based on this finding, we recommend that iron and phosphorus supplementation be given in the diet of fertile cows to reduce the incidence of repeat breeding.

One recent study in Angus cows showed increased milk production and a decreased rate of pregnancy from artificial insemination after mineral mixture supplementation in the diet during gestation, but the overall pregnancy rate was unaltered, regardless of mineral supplementation [18]. Another randomized controlled trial in overconditioned Holstein cows showed the feasibility of increased rate of pregnancy after the injection of trace mineral mixtures 25 days before estrous synchronization [19]. However, this study did not find any difference between follicular and luteal growth related to trace mineral mixture injection in those cows. Another 90-day trial of chelated mineral mixture supplementation to the diet improved the conception rate in repeat breeder buffaloes [20]. A recent study conducted in Nepal reported that hormone therapy (ovsynch protocol) reduced the incidence of repeat breeding in both heifers and buffaloes, as reflected in an improved conception rate [21]. This study, however, emphasizes the role of nutrition and minerals equally for preventing repeat breeding. A recent review outlined the possible ways to prevent repeat breeding, which includes careful handling of the genitalia during artificial insemination, prompt and proper treatment of uterine infections, hormone therapy, and appropriate artificial insemination technique [22]. One study indicated that oocyte apoptosis/damage due to mitochondrial dysfunction of the cows is associated with repeat breeding during the summer [23]. Anti-sperm antibodies found in the serum of repeat breeder cows may cause agglutination and immobilization in spermatozoa, resulting to failure to fertilize the oocyte [24]. A study found that high lipopolysaccharide concentrations in ovulatory follicles were associated with the incidence of repeat breeding [25]. The same study found that high serum estradiol-17 concentration and high neutrophil count in the cervical fluid at artificial insemination can also result in repeat breeding in dairy cows [25]. Estrous pre-synchronization using prostaglandin and GnRH hormones significantly increased the number of cows with a functional corpus luteum an increase in circulating progesterone concentrations [26]. All of the cited studies on repeat breeding have described its multifactorial nature and that mineral deficiencies and imbalances are a factor, consistent with our findings.

### Limitations and future directions

Our study did not assess breed purity or homogeneity, and the cows lacked hormonal profiling before and after the experiment. Researchers who conduct future, broader screening of other macro- and micronutrients in repeat breeder sera should seek more precise correlations between mineral deficiencies and repeat breeding. Likewise, for practitioners, we call for the diet of repeat breeder cows to be supplemented with iron and phosphorus. The effect of this addition on reducing the incidence of repeat breeding should be examined. The hormonal profiling of certain hormones, such as luteinizing hormone, follicle-stimulating hormone, estrogen, and progesterone, before and after iron and phosphorus supplementation in the diet will also help clarify the relationship between levels of minerals and hormones for fertility.

## Conclusion

This is a novel study for the geographic region of Southeast Asia, in that it investigates the potential role of systemic mineral status, in particular, iron, phosphorus, and total protein, in repeat breeding in crossbred Holstein Friesian cows. This research found that repeat breeding in these cows was directly correlated with significantly lower systemic levels of iron and phosphorus but was independent of the systemic total protein level. The findings of this research may prompt farmers to increase the level of minerals in the diet of their repeat breeder cows and to check the levels of these minerals and of protein in the feed and the soil where the grain and grass that they feed their livestock are grown. However, additional comprehensive research on a broad spectrum of hormones and minerals is warranted.

## Authors’ Contributions

GR designed and conducted the experiment. IPD supervised the study. GR drafted the manuscript and interpreted the data. GR and IPD edited and revised the manuscript. Both authors read and approved the final manuscript.
